# The Expression and Amplification of HER2 Has a Significant Impact on the Prognosis of Endometrial Carcinoma in Korean Patients

**DOI:** 10.3390/jcm13082158

**Published:** 2024-04-09

**Authors:** Wook Youn Kim, Eun Jung Yang, Eun Bi Jang, A Jin Lee, Kyeong A So, Seung-Hyuk Shim, Tae Jin Kim, Sun Joo Lee

**Affiliations:** 1 Department of Pathology, KonKuk University Hospital, Konkuk University School of Medicine, 120-1 Neungdong-ro, Hwayang-dong, Gwangjin-gu, Seoul 05030, Republic of Korea; 20100182@kuh.ac.kr; 2 Department of Obstetrics and Gyneacology, Soonchunhyang University Cheonan Hospital, Cheonan 31151, Republic of Korea; 117652@schmc.ac.kr; 3 Department of Obstetrics and Gynaecology, KonKuk University Hospital, Konkuk University School of Medicine, 120-1 Neungdong-ro, Hwayang-dong, Gwangjin-gu, Seoul 05030, Republic of Korea; 20180117@kuh.ac.kr (E.B.J.); 20170050@kuh.ac.kr (A.J.L.); 20190001@kuh.ac.kr (K.A.S.); 20130131@kuh.ac.kr (S.-H.S.); 20190002@kuh.ac.kr (T.J.K.)

**Keywords:** endometrial carcinoma, HER2, immunohistochemistry, silver in situ hybridization, survival outcome

## Abstract

**Objective**: The purpose of this study was to analyze the protein overexpression and gene amplification of HER2 in endometrial carcinoma (EC) and to evaluate its role as a prognostic factor in Korean women. **Methods**: A tissue microarray (TMA) was constructed from samples from 191 patients with diverse histologic types of EC. HER2 protein expression and gene amplification status were analyzed using immunohistochemistry (IHC) and silver in situ hybridization (SISH), respectively. All patients were treated and followed up at a single tertiary medical center in Seoul, Korea, between July 2009 and October 2020. **Results**: In terms of histological type, among the 191 EC patients, 157 had endometrioid carcinoma, nine had uterine serous papillary carcinoma (USPC), one had clear cell carcinoma, one had squamous cell carcinoma, eight had mixed carcinoma, and 15 had uterine carcinosarcoma (UC). HER2 protein overexpression was observed in eight of the 191 (4.2%) EC patients; of these patients, five had IHC scores of 2+, and three had IHC scores of 3+. The HER2 overexpression rates of USPC, UC, and endometrioid carcinomas were 33.3%, 26.6%, and 0.6%, respectively. HER2 protein overexpression was significant in USPC and UC tissues (*p* < 0.000) and was associated with poor overall survival (OS) (*p* < 0.001). *HER2* gene amplification was confirmed in seven of 184 patients (3.8%), including three patients with USPC and four patients with UC. OS was significantly shorter in patients who had HER2 amplification (*p* < 0.001). On multivariate analysis, HER2 expression and *HER2* amplification were statistically significantly associated with worse OS (*p* = 0.006). However, HER2 expression without amplification was not statistically associated with OS (*p* = 0.993). **Conclusions**: HER2 protein overexpression and gene amplification are significantly correlated with shorter OS in Korean women. HER2 can be considered an important predictor of survival outcomes in EC patients.

## 1. Introduction

In South Korea, endometrial carcinoma (EC) was estimated to be the ninth most common cancer among women and the 14th leading cause of death from cancer in 2022 [1]. The incidence of EC has been increasing continuously in South Korea [2]. EC incidence and survival outcomes differ regionally. The age-standardized incidence and mortality rates in South Korea in 2020 were 7.6 and 0.8, respectively, per 100,000 people [2]. In contrast, the age-standardized incidence and mortality rates in North America/North Europe in 2020 were 21.1/3.1 and 16.4/2.7, respectively, per 100,000 people [3].

Among the histotypes of EC, uterine serous papillary carcinoma (USPC) is an aggressive subtype, accounting for less than 10% of EC cases, and it has a 5-year survival rate of approximately 30% in all stages; more than 50% of relapses and deaths due to EC occur in patients with USPC [4]. Uterine carcinosarcoma (UC) (previously known as malignant mixed Mullerian tumor) is a rare subtype, accounting for approximately 6% of EC cases, but it accounts for 16% of deaths among EC patients [5]. UC consists of carcinoma and sarcoma components, and the epithelial part of UC commonly consists of serous carcinoma in its pure form or mixed with other carcinoma types [5]. UC is a rare and highly aggressive malignancy. EC shows a different distribution of histotypes according to racial background. A high proportion of patients with the low-grade endometrioid type are Caucasian, and USPC and UC are more common among African Americans [6]. Among USPC patients, African American patients have a poorer prognosis (*p* = 0.01) and significantly higher rates of human epidermal growth factor receptor 2 (*HER2*) gene amplification than Caucasian women (*p* = 0.02) [7]. In addition, the cumulative mortality rate of EC is greater in African Americans than in patients of other races [6].

HER2 is a cell membrane glycoprotein located on chromosome 17 that encodes a tyrosine kinase receptor and belongs to the epidermal growth factor receptor family. By activating tyrosine kinase, it initiates a signal transduction pathway leading to cell division and functions in signal proliferation, differentiation, and apoptosis inhibition [8]. Upon alteration of genes related to HER2 expression control (e.g., TP53), HER2 is amplified and induces cancer formation. Gene amplification and protein overexpression of HER2 have been shown to play important roles in the pathogenesis of cancers in a variety of organs, including carcinomas of the breast, ovary, stomach, and esophagus [9]. In particular, overexpression and amplification of HER2 were reported in 18–42% of USPC patients [4,6,10] and 14% of UC patients [11]. These phenotypes have also been reported to be associated with a poor prognosis [4].

The purpose of this study was to determine the expression rate of HER2 in Korean women and to confirm its correlation with prognosis. Another goal is to compare these features in Korean women to those of women of other races. In addition, this study was designed to investigate the association between HER2 protein expression, as determined by immunohistochemistry (IHC), and gene amplification, as determined by silver in situ hybridization (SISH), in Korean patients with EC. This information could increase the appropriateness of the evaluation criteria used in IHC and SISH for detecting gene amplification in patients with EC.

## 2. Materials and Methods

### 2.1. Patients

Seoul, the capital city of South Korea, and its surrounding areas are “melting pots” of Korean people who have migrated from all over the country. Our hospital is a tertiary medical center located in Seoul. We retrospectively reviewed the medical records of all patients who were diagnosed with EC and who underwent standard treatment at this institution between July 2009 and October 2020. All patients were identified as Korean, an East Asian ethnicity. Patients of all stages underwent primary surgery and then received adjuvant treatment, as needed, according to the National Comprehensive Cancer Network (NCCN) guidelines (radiotherapy, chemotherapy, or sequential chemotherapy and irradiation). Patients were followed up every 3–6 months for 3 years after treatment and every 6 months thereafter. After 5 years, patients were followed every 1 year. Patients who did not receive the recommended standard treatment for EC or patients for whom tissue slides could not be generated due to the small amount of tissue obtained were excluded. During the study period, 191 patients were enrolled. Of the 191 patients, 154 (80.6%) resided within 20 min of our hospital, and the remaining patients came from various parts of the country. The population of this study was considered to be representative of the Korean population. Clinical data such as patient age, histologic subtype, disease stage, histological grade, myometrial invasion, disease recurrence, progression-free survival (PFS), and overall survival (OS) were analyzed. The surgical stage of all patients was modified according to the 2019 International Federation of Gynecology and Obstetrics and Gynecology (FIGO) staging system. The date of diagnosis was the date when the histopathological diagnosis was confirmed. PFS was defined as the period from the date of diagnosis to the date of recurrence or censoring, and OS was defined as the period from the date of diagnosis to the date of death, last follow-up, or censoring.

The protocol was approved by the institutional review board of Konkuk University Hospital (approval number: KUMC 2020-10-024).

### 2.2. Construction of a Tissue Microarray Using Representative Tissue Samples

Representative tissue samples were taken from 191 uterine ECs to construct the tissue microarray (TMA). Tissue cores (3 mm in diameter) were obtained from formalin-fixed, paraffin-embedded tissue blocks of 191 EC patient donors and arranged on recipient TMA blocks [12]. The TMA slides were histologically reviewed by WYK to confirm the diagnosis of the corresponding tumor tissues in each TMA spot after hematoxylin and eosin staining.

### 2.3. Immunohistochemistry

The 3 µm thick sections of TMA blocks were immunostained with a rabbit anti-HER2/neu (4B5) monoclonal antibody (Ventana Medical Systems, Tucson, AZ, USA) using an autoimmunostainer (BenchMark ULTRA, Ventana Medical Systems) [13]. An OptiView DAB detection kit (Ventana Medical Systems) was used for the detection of immunoreactions against HER2 [14]. In addition, HER2-positive breast cancer tissues were immunostained in parallel as a positive control.

Two pathologists (WYK and JHP) independently performed the immunohistochemical staining. Discrepant results in controversial cases were reviewed and determined in a common session using a multiview microscope.

HER2 protein expression on TMA blocks was evaluated by a four-tier system based on the DAKO HercepTest guidelines (DAKO, Glostrup, Denmark) [13,15]. The immunohistochemical staining was scored as follows: 0, no staining or membrane staining in less than 10% of the tumor cells; 1+, faint/barely perceptible membrane staining in >10% of the tumor cells; 2+, weak-to-moderate complete membrane staining in more than 10% of the tumor cells; and 3+, strong complete membrane staining in more than 10% of the tumor cells [13,15]. Scores of 2+ and 3+ were considered to indicate positive HER2 expression.

### 2.4. Dual-Color SISH

Copy number alterations in the *HER2* gene were evaluated by automated dual-color SISH using a Ventana BenchMark GX (Ventana Medical Systems). SISH signals of the *HER2* gene were detected using an INFORM *HER2* DNA Probe (Ventana Medical Systems) and an UltraView SISH Detection Kit (Ventana Medical Systems) [16]. The centromere of chromosome 17 (CEP 17) was visualized via the digoxigenin (DIG)-labeled Chromosome 17 Probe (Ventana Medical Systems) and the UltraView Red DIG Detection Kit (Ventana Medical Systems) [13].

SISH signals were evaluated according to the 2016 American Society of Clinical Oncology (ASCO)/College of American Pathologists (ASCO-CAP) guidelines for gastric cancer. *HER2* gene copy number was assessed in the 20 cohesive tumor cells showing the highest gene count. *HER2* gene amplification was considered positive in patients with a *HER2*/CEP17 ratio ≥ 2.0 or *HER2* polysomy (ratio < 2.0 and *HER2* signal > 6.0 per nucleus). In addition, clustered multiple signals were scored according to the interpretive guide for Ventana INFORM *HER2* DNA probe staining of breast carcinoma (Ventana Medical Systems) [17]. Specifically, a small cluster and large cluster were interpreted as 6 signals and 12 signals, respectively. SISH staining was independently scored by two pathologists (WYK and JHP), and the pathologists reached a consensus on any controversial results in a common session using a multiview microscope.

### 2.5. Statistical Analysis

All the statistical analyses were performed using SPSS version 22.0 (IBM SPSS Statistics, Chicago, IL, USA). The frequency distributions were analyzed using the chi-square test and Fisher’s exact test. The Jonckheere test was performed to evaluate the association between increased HER2 protein expression or *HER2* gene amplification, and for multiple comparisons between histologic subtype groups, the least significant difference test was performed. Multivariate survival analysis was performed using the Cox proportional hazards model to examine the association between surgical-pathological variables and outcome. Correlations between IHC and SISH results were evaluated using Pearson correlation analysis. PFS and OS were assessed using Kaplan-Meier survival analysis, and the results were compared using log-rank tests. A *p* value < 0.05 was considered to indicate statistical significance.

## 3. Results

### 3.1. Clinical Data

**Figure 1:**
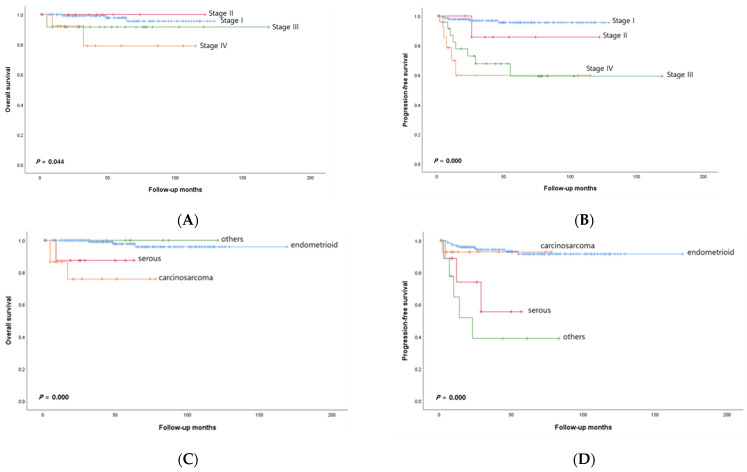

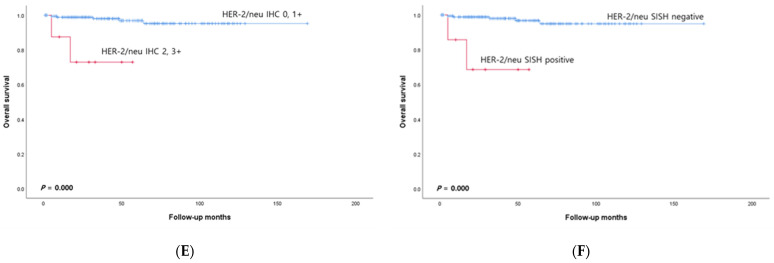
(**A**–**F**), Kaplan-Meier curves for OS and PFS in the enrolled patients with endometrial carcinoma. OS and PFS for patients according to FIGO stage (**A**,**B**) and histological subtype (**C**,**D**). OS curves for patients with endometrial carcinoma stratified by (**E**) HER2 overexpression detected by IHC and (**F**) positivity of SISH for amplification of the HER2 gene.

Of the 191 EC patients included in this study, 157 had endometrioid carcinoma, nine had UPSC, one had clear cell carcinoma, one had squamous cell carcinoma, eight had mixed carcinoma, and 15 had UC. Table 1 presents the clinicopathological features of the enrolled patients. Of all the patients, 102 patients received adjuvant treatment after surgery. Forty patients received systemic chemotherapy, 44 patients received radiation therapy, and 18 patients received sequential chemotherapy and irradiation. Eighty-nine patients were in the early stage and did not receive postoperative treatment. Fifty-nine patients (30.9%) were at high risk for EC according to the Gynecologic Oncology Group criteria. Nineteen patients (9.9%) experienced relapse, and seven patients (3.7%) died of recurrent disease during the study period. Among the patients who died, three patients (42.8%) had UC, three patients (42.8%) had endometrioid carcinoma, and one patient (14.4%) had UPSC. Figure 1A,B illustrates the survival results according to FIGO stage. The patients grouped by FIGO stage exhibited statistically significant differences in OS and PFS (*p* = 0.044 and *p* = 0.000, respectively). UC patients had worse OS than patients with all other cell types (*p* = 0.000; Figure 1C). However, patients with USPC had significantly worse PFS than patients with other cell types (*p* = 0.000; Figure 1D).

### 3.2. HER2 Protein Expression Analysis via IHC

**Figure 2:**
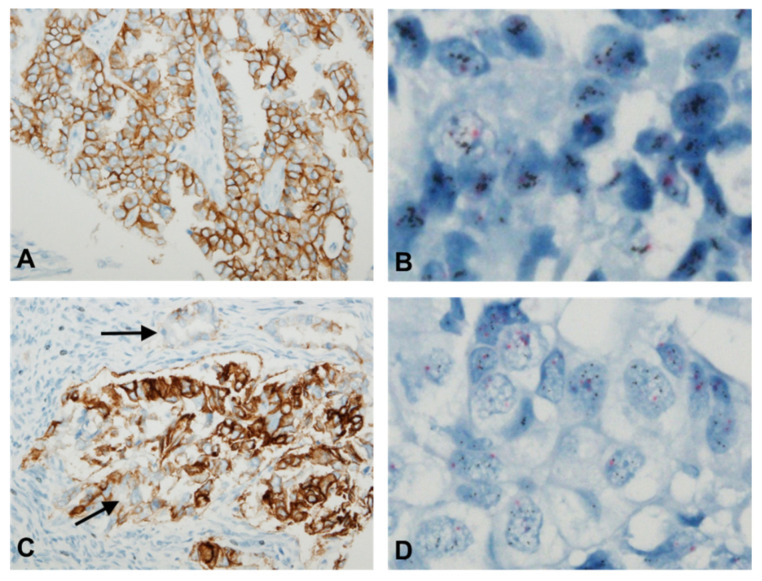
Representative findings of HER2 IHC and SISH in uterine endometrial carcinomas. (**A**,**B**): A patient with uterine serous carcinoma with an IHC score of 2+ (**A**) and *HER2* gene amplification by SISH (**B**) is shown. (**C**,**D**): Representative images of uterine carcinosarcoma tissue samples showing an IHC score of 3+ (**C**) and *HER2* gene amplification (**D**) are shown. IHC indicated that this carcinosarcoma case exhibited significant intratumoral heterogeneity of HER2 expression, with coexisting tumor cells showing IHC scores of 1+ and 2+ (arrows) in this area, which was also correlated with a heterogeneous pattern of *HER2* amplification.

HER2 protein expression was evaluated using IHC in 191 patients; 183 (95.9%), 5 (2.6%), and 3 (1.6%) patients had scores of 0/1+, 2+, and 3+, respectively (Table 2). Table 3 shows the detailed clinicopathological characteristics of patients with HER2 protein overexpression and gene amplification. Representative findings of HER2 IHC in USPC (Figure 2A) and UC (Figure 2C) are shown. A statistically significant difference in HER2 expression was confirmed across the histological subtypes (*p* < 0.0001; Table 2). IHC scores of 2+/3+ were confirmed in 33.3% of USPC patients and 26.6% of UC patients. Among the histological subtypes, serous cancer had the highest proportion of samples with IHC scores of 2+/3+ (*p* < 0.0001; Table 2). Patients with EC with HER2 overexpression had a significantly shorter OS than did patients with cancer without HER2 overexpression (*p* = 0.000; Figure 1E). However, there was no significant difference in PFS according to HER2 overexpression status (*p* = 0.423).

### 3.3. HER2 Gene Amplification Analysis via SISH

*HER2* gene amplification was detected in 7 of 184 patients (3.8%) via SISH. Seven specimens failed to be TMA-prepared for SISH testing. Positive *HER2* SISH results were detected in three patients with USPC (33.3%) and four patients with UC (28.6%). However, no other histotypes were found to be *HER2* SISH positive (Table 2 and Table 3). Representative findings of *HER2* gene amplification in USPC (Figure 2B) and UC (Figure 2D) are shown. There were statistically significant differences in *HER2* gene amplification status across the histological subtypes (*p* < 0.0001; Table 2). Patients positive for *HER2* SISH had significantly worse OS (*p* = 0.000; Figure 1F). However, there was no difference in PFS between patients with *HER2*-positive and *HER2*-negative tumors according to SISH (*p* = 0.459).

### 3.4. Relationships between Surgical-Pathologic Variables and Survival Outcome

Univariate analysis (Table 4) showed that advanced stage (stage III and IV) and lymph-vascular space invasion correlated with shorter PFS. HER2 expression and *HER2* amplification showed a marginal correlation with shorter OS (*p* = 0.049). However, in the multivariate model (Table 5), HER2 expression and *HER2* amplification were statistically significantly associated with worse OS (*p* = 0.006). HER2 expression without amplification was not statistically associated with OS (*p* = 0.993). Although advanced stage (stage III and IV) and lymph-vascular space invasion correlated with shorter PFS (*p* = 0.006 and *p* = 0.020, respectively), HER2 expression and *HER2* amplification did not correlate with PFS (*p* = 0.985). 

### 3.5. Associations between HER2 Protein Expression and Gene Amplification

There was a significant correlation between high HER2 protein expression and gene amplification (*r* = 0.411, *p* < 0.000). Of the 183 (95.9%) patients with negative *HER2* expression, 177 (96.2%) were negative for *HER2* amplification, according to SISH. In contrast, of the eight patients with positive HER2 expression, seven had gene amplification, according to SISH. Patients who were positive for *HER2* amplification according to SISH tended to have high IHC scores.

## 4. Discussion

This study included all EC patients diagnosed and treated after July 2009 at a single institute without selection. Moreover, 80.6% of the patients were considered direct relatives, many of whom emigrated from all over the country. Therefore, the patients included in this study are believed to represent the general population of Korea.

Santin, A.D. et al. reported that the prognosis differed according to race in patients in the USPC. Compared with Caucasian (C) patients, African American (AA) patients with USPC have been shown to have a worse prognosis (5-year OS, 18.0% vs. 67.0%) [7]. The 5-year USPC survival rate of the Korean women in this study was 87.5%, which was superior to that of the C patients (67.0%) and AA patients (18.0%). Among USPCs, 33.3% had HER2 protein overexpression, and 33.3% had *HER2* gene amplification. The *HER2* gene amplification rates in C and AA patients were 33% and 67%, respectively [7]. The amplification of the *HER2* gene in Koreans was similar to that in C patients and lower than that in AA patients. In USPC, differences in prognosis between races are likely related to *HER2* gene amplification.

Among the subtypes of EC, the incidence of UC in Koreans was 7.9%, which was higher than that in a previous study (6%) [5]. The epithelial component of UC generally comprises serous carcinoma, and it has been reported in previous studies that these serous components are confirmed to be positive for HER2 overexpression [9]. In the present study, HER2 protein overexpression and *HER2* gene amplification were identified in serous carcinoma specimens (Table 3). In this study, the percentages of UC patients with HER2 protein overexpression and gene amplification were confirmed to be 26.6% and 28.6%, respectively. The percentage of Koreans with HER2-positive UC was greater than that in Amant et al.’s study (14%) [18]. Therefore, the evaluation of HER2 status in UC patients should be considered in Korean women.

In the present study, we evaluated IHC (to assess HER2 protein expression) [13] and SISH (to assess *HER2* gene amplification) using the criteria for breast cancer. However, it is not yet clear whether the criteria for breast cancer are appropriate for uterine cancer. It is also unclear whether SISH is more appropriate than fluorescence in situ hybridization (FISH) or chromogenic in situ hybridization (CISH) for gene amplification confirmation. Even in breast cancer patients, previous studies have shown obvious discrepancies between protein expression and gene amplification detected via FISH [15,19]. Moreover, Grazziotin et al. reported that the agreement between bright field test results (SISH and CISH) and FISH results was high (≥92%) [20]. CISH was reported to perform better than SISH [20]. However, we identified a significant correlation between increased *HER2* gene amplification and high HER2 protein expression (*r* = 0.411, *p* < 0.000). Except for one patient with endometrioid adenocarcinoma, which exhibited only HER2 overexpression, all patients with UC with a serous component or USPC had concordant HER2 IHC and *HER2* SISH results (Table 3). The present study suggested that the methods we used are appropriate because the features detected are highly correlated with patient prognosis. In addition, the high performance of SISH for the assessment of *HER2* gene amplification was demonstrated in this study. 

In the present study, 2.8% (5/177) of HER2-negative EC patients died, whereas 28.6% (2/7) of HER2-positive EC patients died. These findings suggest that HER2 positivity may be a predictive biomarker for poor prognosis. However, HER2 expression and amplification were statistically significantly associated with worse OS (*p* = 0.006) but not with worse PFS (*p* = 0.982) on multivariate analysis. As PFS is primarily an assessment of disease progression, it only considers time to progression. Therefore, differences in PFS may be determined by the rate of disease progression. However, OS includes both disease progression and death from other causes, so longer follow-up may reveal statistically significant differences. In addition, although EC recurrence is multifactorial, it is reasonable to assume that HER2-positivity has a significant impact on survival. 

HER2 can be a treatment target. Trastuzumab, a monoclonal antibody against HER2, is used as a treatment for HER2-positive cancer patients. Fader et al. reported an increase in PFS and OS when trastuzumab was added to the carboplatin–paclitaxel regimen in patients with HER2-positive, metastatic, and advanced uterine USPC [21]. Jenkins et al. reported that UC typically lacks MMR deficiency and/or strong PD-L1 expression. Therefore, UC does not respond well to treatment with immune checkpoint inhibitors [22]. UC and USPC accounted for 42.8% of EC-related deaths in Koreans, so treatment targeting HER2 can be considered. Nevertheless, USPCs are predominantly of the p53abn/copy number high molecular subtype and have a strong correlation between abnormal *TP53* status and *HER2*. Therefore, molecular classification may be used in the future to identify patients who may benefit from anti-HER2 agents [23]. 

This study has several limitations. First, it is a retrospective study of a relatively small number of patients with EC, and these factors may have biased the results. Second, we believe that this study was representative of the general population of Korean women. This may be a misconception, and a more sophisticated study design is needed for verification. Thirdly, TMA preparation for SISH testing was not possible for seven specimens, probably due to poor preservation. Finally, we were unable to include specimens collected from many UC patients before the study period because TMA preparation was not possible for older specimens. This may have been a source of selection bias in this study. Fourthly, *TP53* mutation was not identified in this study. The Cancer Genome Atlas (TCGA) confirmed that *TP53* mutation and high frequency of *HER2* amplification are characteristic of UPSC [24,25]. Ross et al. reported that *HER2* amplification and *TP53* mutation/high grade histology often co-express [26]. Progesterone receptor has also been reported to be one of the strongest prognostic factors [27], which we did not confirm in this study, and it is believed that this may also act as a bias in determining the prognostic impact of HER2. As a result, this study did not identify other molecular prognostic factors, which may have further strengthened the prognostic impact of HER2.

In conclusion, Korean women with USPC and UC have high rates of HER2 protein overexpression and gene amplification, and these features are significantly correlated with a poor prognosis. The prevalence of HER2 protein overexpression and gene amplification is similar for Koreans and Caucasians but the prevalence is lower in Koreans than in African Americans. A significant correlation between HER2 positivity and a poor prognosis suggested that anti-HER2 therapy may be an option for treating EC. Nevertheless, due to the small sample size and number of events, further studies are needed to draw definitive conclusions.

## Figures and Tables

**Table 1:** Clinical characteristics of the enrolled patients with endometrial cancer.

Parameter	Number of Patients (%) (*n* = 191)
Age, n (%)	
<50	67 (35.1)
≥50	124 (64.9)
Histologic subtype, n (%)	
Endometrioid	157 (82.2)
Serous	9 (4.7)
Clear cell	1 (0.5)
Squamous	1 (0.5)
Mixed	8 (4.2)
Carcinosarcoma	15 (7.9)
Stage of disease, n (%)	
I	142 (74.3)
II	10 (5.2)
III	24 (12.6)
IV	15 (7.9)
Histological grade, n (%)	
1	84 (44.0)
2	66 (34.6)
3	26 (13.6)
unknown	15 (7.8)
Myometrial invasion, n (%)	
<1/2	133 (69.6)
≥1/2	58 (30.4)
Adnexal involvement, n (%)	
No	171 (89.5)
Yes	20 (10.5)
Lymph-vascular space invasion, n (%)	
No	145 (75.9)
Yes	46 (24.1)
Lymph node involvement	
No	166 (86.9)
Yes	25 (13.1)
Recurrence of disease, n (%)	19 (9.9)
Died of disease, n (%)	7 (3.7)

**Table 2:** HER2 overexpression and gene amplification in endometrial carcinomas.

Histotype	HER2 IHC			*p*	*HER2* SISH		*p*
	Negative 0 or 1+	Positive			Negative	Positive	
		2+	3+				
Endometrioid	156 (99.4)	1 (0.6)	0	<0.0001	151 (100.0)	0	<0.0001
Serous	6 (66.6)	2 (22.2)	1 (11.1)		6 (66.7)	3 (33.3)	
Clear cell	1 (100.0)	0	0		1 (100.0)	0	
Squamous	1 (100.0)	0	0		1 (100.0)	0	
Mixed	8 (100.0)	0	0		8 (100.0)	0	
Carcinosarcoma	11 (73.3)	2 (13.3)	2 (13.3)		10 (71.4)	4 (28.6)	
Total	183 (95.9%)	5 (2.6%)	3 (1.6%)		177 (96.2%)	7 (3.8%)	

Abbreviations: IHC, immunohistochemistry; SISH, silver in situ hybridization.

**Table 3:** Tumor types, clinical data, HER2 expression and gene amplification status.

Histologic Type	Age	Stage	Carcinoma Type	Sarcoma Type	HER2 IHC	*HER2* SISH
Carcinosarcoma	64	IVB	Serous	Rhabdomyosarcoma	3+	Positive
Carcinosarcoma	57	IVA	Serous	Homologous	3+	Positive
Carcinosarcoma	83	IA	Serous	Homologous	2+	Positive
Carcinosarcoma	65	IB	Serous	Homologous	2+	Positive
Serous	59	IA	-	-	3+	Positive
Serous	62	IA	-	-	2+	Positive
Serous	63	IIIC2	-	-	2+	Positive
Endometrioid	69	IB	-	-	2+	Negative

**Table 4:** Univariate analysis.

Factors	N	Overall Survival			Progression-Free Survival		
		HR	95% CI	*p*	HR	95% CI	*p*
Age							
<50	67	1.00			1.00		
≥50	124	2.27	0.20–25.41	0.505	2.10	0.66–6.72	0.210
Histology							
Endometrioid	157	1.00			1.00		
Nonendometrioid	34	0.98	0.07–13.06	0.988	1.64	0.46–5.84	0.440
Grade							
Endometrioid G1, G2	145	1.00			1.00		
Endometrioid G3, serous, clear cell, carcinosarcoma	46	3.44	0.24–48.39	0.359	3.02	0.78–11.69	0.110
Stage of disease							
I or II	152	1.00			1.00		
III or IV	39	1.00	0.09–10.82	1.000	3.34	0.89–12.47	0.043
Myometrial invasion,							
<1/2	133	1.00			1.00		
≥1/2	58	3.23	0.39–26.86	0.278	0.57	0.19–1.73	0.323
Adnexal involvement							
No	171	1.00			1.00		
Yes	20	4.49	0.46–43.64	0.195	1.28	0.43–3.83	0.663
Lymph-vascular space invasion							
No	145	1.00			1.00		
Yes	46	0.89	0.09–8.25	0.921	4.41	1.34–14.55	0.015
HER2 IHC/HER2 SISH							
Negative/negative	183	1.00					
Positive/negative	1	0.00	0.00	0.994	0.00	0.00	0.996
Positive/positive	7	7.86	1.85–72.28	0.049	0.00	0.00	0.985

**Table 5:** Multivariate analysis.

Factors	N	Overall Survival			Progression-Free Survival		
		HR	95% CI	*p*	HR	95% CI	*p*
Age							
<50	67	1.00			1.00		
≥50	124	2.42	0.27–22.00	0.434	2.08	0.688–6.290	0.194
Stage of disease							
I or II	152	1.00			1.00		
III or IV	39	3.44	0.05–23.36	0.206	5.00	1.58–15.79	0.006
Lymph-vascular space invasion							
No	145	1.00			1.00		
Yes	46	2.24	0.32–15.84	0.419	3.94	1.24–12.55	0.020
HER2 IHC/HER2 SISH							
Negative/negative	183	1.00					
Positive/negative	1	0.00	0.00	0.993	0.00	0.00	0.997
Positive/positive	7	12.99	2.08–81.03	0.006	0.01	0.00	0.982

## Data Availability

No new data were created or analyzed in this study. Data sharing is not applicable to this article.
